# Does Addition of Tobramycin Powder Reduce Infection Rates After Spine Surgery?

**DOI:** 10.1177/2192568218776609

**Published:** 2018-05-17

**Authors:** Yu-Po Lee, Saifal-Deen Farhan, Arif Pendi, Torin J. Cunningham, P. Douglas Kiester, Peter Hahn, Charles D. Rosen, Nitin Bhatia

**Affiliations:** 1 University of California, Irvine, Orange, CA, USA; 2 Miller Children’s Hospital, Long Beach, CA, USA

**Keywords:** vancomycin, tobramycin, antibiotic powder, prophylaxis, surgical site infection, instrumented fusion surgery, retrospective chart review

## Abstract

**Study Design:**

Retrospective chart review.

**Objectives:**

To evaluate the efficacy of tobramycin and vancomycin powder in reducing surgical site infections in posterior lumbar instrumented fusion.

**Methods:**

A multicenter, electronic database search was conducted for all patients who underwent posterior instrumented lumbar fusions.

**Results:**

The addition of vancomycin powder decreased postoperative infections from an incidence of 5.7% down to a rate of 2.0%. This difference was statistically significant (*P* = .018). The addition of tobramycin powder to the wound in addition to vancomycin further decreased the infection rate down to 1.8%. The postoperative infection rate was statistically significant (*P* = .041) when compared with the no-powder group. However, the difference was not statistically significant (*P* = 1.00) when compared with vancomycin alone. There was also a trend toward gram-negative organisms with the addition of more antibiotic powder. In the control group, for example, the organisms cultured were 66% methicillin-sensitive *Staphylococcus aureus* and 33% gram-negative organisms. In the vancomycin group, 30% of the organisms cultured were *Staphylococcus aureus* and 60% gram-negative organisms. In the vancomycin and tobramycin powder group, 100% of the organisms cultured were gram-negative.

**Conclusions:**

There is a reduction in surgical site infections with addition of antibiotic powder to the wound prior to closure. However, the reduction in the infection rate was not as great with the addition of tobramycin powder to vancomycin alone and there was a noticeable change in the spectrum of organism cultured with this addition. Clinicians should consider the risk-to-benefit ratio in each case when deciding to use antibiotic powder.

## Introduction

Surgical site infections (SSIs) are a potential complication after spinal surgery. The infection rates of spinal surgeries reported in the literature range from 0.7% to 11.9% depending on the diagnosis and the complexity of the procedure.^1–3^ SSIs account for enormous medical, social, and economic costs for patients as well as hospitals.^4,5^ Direct costs include a longer hospital stay, additional procedures to eradicate the infection, and antibiotics. A postoperative infection may also have an emotional impact on a patient’s view of the overall outcome, despite a generally successful treatment of the infection.

The evidence suggests that systemic intravenous antibiotic prophylaxis reduces the risk of postoperative infections.^6–8^ Cephalosporins are widely used, based on their good efficacy against staphylococcal species and uropathogens.^6^ Vancomycin is indicated in high-risk patients carrying methicillin-resistant *Staphylococcus aureus*.^6^ If the patient has allergies to β-lactam antibiotics, clindamycin or vancomycin can be used.

It should be noted that antibiotics may have a limited penetrance into disc tissue even if appropriately dosed. In a study by Walters et al,^9^ the authors evaluated the antibiotic levels in the annulus and nucleus pulposus in a sheep model. Cephazolin was given 30 minutes prior to sacrifice. It was noted that antibiotic levels were greater in the annulus than in the nucleus pulposus. They concluded that while the incidence of iatrogenic discitis can be reduced by antibiotic prophylaxis, it could not be abolished in all incidences with a broad-spectrum antibiotic such as cephazolin. To counteract inadequate antibiotic penetrance and possible iatrogenic seeding of the surgical site, some studies have evaluated the use of vancomycin powder in the surgical site.^10,11^ In one study by O’Neill et al,^10^ the authors performed a retrospective review on 110 patients who underwent a posterior instrumented fusion for thoracolumbar fractures. There were no infections in the group where vancomycin powder was added to the wound while they noted a 13% infection rate in cases where they only used standard antibiotic prophylaxis. This difference was noted to be statistically significant. In another study by Sweet et al,^11^ the authors performed a retrospective review on 1732 consecutive thoracic and lumbar posterior instrumented spinal fusions. They noted a 2.6% deep infection rate in patients who received standard antibiotic prophylaxis while the addition of 2 g of vancomycin power decreased the infection rate to 0.2%.

Hence, the use of vancomycin powder has increasing evidence to support its use as well. But there are incidences of infections even with the use of vancomycin powder.^12,13^ In a study by Tubaki et al,^12^ the authors performed a prospective, randomized controlled study on 907 patients undergoing spinal fusion. The authors noted 8 (1.68%) infections in the control group, which received no powder while they noted 7 (1.61%) infections in the treatment group which received vancomycin powder. Of note, they cultured 3 organisms in the control group (1 *Escherichia coli* and 2 *Staphylococcus aureus*). In the treatment group, they cultured 3 organisms as well (1 *Staphylococcus aureus* and 2 *Klebsiella*). This led the authors to conclude that the intraoperative use of vancomycin powder was not effective in reducing infection rates in spinal surgery. Of concern is that it may be selecting for gram negative organisms. So, this has led some surgeons to use tobramycin powder in addition to vancomycin powder in complex cases. To date, this has not been fully studied in spinal surgery and the purpose of this study is to evaluate if the addition of tobramycin powder to vancomycin powder reduces the infection rate.

## Materials and Methods

The current study is an institutional review board–approved retrospective evaluation of patients undergoing spinal surgery between August 2010 and December 2015 at the University of California San Diego, Miller Children’s Hospital and University of California Irvine. As this was a retrospective study, a waiver of consent was request and granted by the institutional review board.

A retrospective review of patients presenting with spinal diseases requiring surgery was performed. This included patients with disc herniations, stenosis, spondylolisthesis, spinal deformity, and traumatic injuries. Patients who underwent a lumbar decompression and/or posterior instrumented fusion were included in the study. All patients had a standard chlorhexidine prep with 2 g of intravenous cefazolin given within an hour before incision and continued for 24 hours. (Patients at Miller Children’s were given intravenous cefazolin based on weight as well as intravenous tobramycin.) Data recorded include their initial diagnosis, procedure performed, whether antibiotic powder was placed into the wound, and complications. Other data collected included age, gender, medical comorbidities (hypertension, cardiac disease, diabetes, or osteoporosis), and occupation. Patients were then divided into 1 of 3 groups based on the type (or lack) of antibiotic powder(s) used intraoperatively. The antibiotic powder regimen employed was left up to surgeon discretion. In the vancomycin group, 1 to 2 g of vancomycin were placed into the wound on closure (1 g for pediatric patients and 2 g for adults). In the vancomycin and tobramycin group, 2 g of vancomycin and 1 g of tobramycin was placed into the wound. A condition was considered a significant medical comorbidity if it was listed in the patient’s past medical history. In addition, social history, such as smoking and alcohol use, was also evaluated. Patients older than 75 years were excluded from the study.

If a wound infection was suspected based on clinical examination and laboratory results (complete blood count with differential, erythrocyte sedimentation rate, C-reactive protein), then the wound was explored such as under general anesthesia if deep wound infection was suspected. Aerobic, anaerobic, acid fast bacilli, and fungal cultures were obtained. The wounds were classified and treated according to the depth of infection. Superficial infections involved the superficial skin or subcutaneous tissues and were treated with local wound care and 5 to 7 days of oral antibiotics. Deep wound infections were those involving the subfascial layers and the spinal instrumentation and treated with serial surgical debridement intravenous antibiotics, and consultation with infectious disease specialists.

### Statistical Methods

Fisher’s exact test was used to determine if there was a significant difference in infection rates between the nonantibiotic group versus vancomycin powder and vancomycin with tobramycin groups, respectively. A chi-square test was performed to see if there was a difference between age, alcohol use, smoking, and medical comorbidities among the groups. A logistic regression analysis was also performed to see if these factors were independent risk factors for developing infection after surgery. All hypothesis testing was performed with a level of significance of .05.

## Results

There were 209 patients in the control group where no antibiotic powder was added, 489 patients in the vancomycin powder group, and 219 patients in the vancomycin and tobramycin powder group. The age of patients included in this study ranged from 7 to 75 years. The average age of the patients in the no powder group was 56 years old, the average age of the vancomycin group was 42 years, and the average age of the vancomycin and tobramycin group was 61 years old. In the no powder group, there were 63.5% females and 36.5% males. In the vancomycin group, there were 55.5% females and 44.5% males. In the vanocmycin and tobramycin group, there were 61.3% females and 38.7% males. The number of levels fused and the number of nonfusion decompression were comparable across the vancomycin and tobramycin group, vancomycin alone group, and control group.

### Infection Rate

There were 12 infections out of 209 cases in the no powder group (5.7%) (Table 1). There were 10 infections out of 489 cases in the vancomycin group (2.0%). There were 4 infections out of 219 cases in the vancomycin and tobramycin group (1.8%). Statistical analysis using Fisher’s exact test was statistically significant (*P* = .018) when comparing the no powder group to the vancomycin group and when comparing the no-powder group to the vancomycin and tobramycin group (*P* = .041). The difference was not statistically significant (*P* = 1.00) when comparing the infection rates of the vancomycin powder group and the vancomycin and tobramycin powder group.

**Table 1.** Surgical Site Infection and Microorganism Culture.

Antibiotic Powder	SSI, n (%)	Total Patients, n	Microorganism culture (n)
Vancomycin and tobramycin	4 (1.8)	219	*Pseudomonas aeruginosa* (2) *Escherichia coli* (1) Coagulase negative *Staphylococcus*, *Klebsiella pneumoniae* (1)
Vancomycin	10 (2.0)	489	MSSA (3) *Pseudomonas aeruginosa* (3) *Streptococcus* group B (1) *Pseudomonas aeruginosa* and *Klebsiella pneumoniae* (1) *Serratia marcescens*, *Pseudomonas aeruginosa*, *Proteus mirabilis*, *Enterococcus*, *Candida parapsilosis* (1)
None	12 (5.7)	209	MSSA (8) *Serratia marcensans* (1) *Enterobacter cloacae* (1) *Klebsiella pneumoniae* (1) *Pseudomonas aeruginosa* (1)

Abbreviations: MSSA, methicillin-sensitive *Staphylococcus aureus*; SSI, surgical site infection.

### Organisms Cultured

The organisms cultured from the no powder group were 8 cases of methicillin-sensitive *Staphylococcus aureus* (MSSA), 1 case of *Serratia marcescens*, 1 case of *Enterobacter cloacae*, 1 case of *Klebsiella pneumoniae*, and 1 case of *Pseudomonas aeruginosa* (Table 1).

The organisms cultured from the vancomycin group were 3 cases of *Staphylococcus aureus*, 3 cases of *Pseudomonas aeruginosa*, 1 case of group B *Streptococcus*, 1 case of *Serratia marcescens*, and 2 cases of multiple gram-negative organisms.

The organisms cultured form the vancomycin and tobramycin powder group were 1 case of *Escherichia coli*, and 2 cases of *Pseudomonas aeruginosa*, and 1 case of mixed MSSA and *Klebsiella pneumoniae*.

### Smoking

To normalize the groups as best as we could, we performed a chi-square analysis to see if there was a disproportionate number of smokers between the groups (Table 2). There were no significant differences between the groups with regard to smokers (*P* = .48).

**Table 2.** Demographic Data, Social History, and Comorbidity.

Antibiotic Powder (n)	Age >50 years, n (%)	Female, n (%)	Alcohol, n (%)	Smoking, n (%)	Comorbidity, n (%)
Vancomycin and tobramycin (209)	169 (77.2)	136 (61.3)	81 (37.0)	59 (27.2)	26 (12.4)
Vancomycin (489)	245 (50.1)	274 (55.5)	54 (11.0)	65 (13.3)	38 (7.7)
None (219)	157 (75.1)	133 (63.5)	71 (17.7)	40 (19.2)	12 (5.7)

In this study, smoking was noted to be a risk factor for developing a postoperative infection. The difference in the infection rates between smokers and nonsmokers as a risk factor for postoperative infections was statistically significant (*P* < .00 001).

### Medical Comorbidities

A chi-square analysis was performed to see if there were a disproportionate number of patients with diabetes and medical comorbidities between the groups. There were no significant differences between the groups with regard to patients with medical comorbidities (*P* = .32).

In this study, the difference in the infection rates between patients with medical comorbidities and those without was not found to be statistically significant but it did approach significance (*P* = .07).

### Age

A chi-square analysis was performed to see if there was a difference in age between the groups. There were no significant differences between the groups with regard to age (*P* = .32).

In this study, the difference in the infection rates between patients older than 65 years and those younger than 65 years was not found to be statistically significant, but it did approach significance (*P* = .07).

### Alcohol

We performed a chi-square analysis to see if there was a disproportionate number of who drank alcohol between the groups. There were no significant differences between the groups with regard to drinkers (*P* = .48).

In this study, alcohol use was not a separate risk factor of infection. The difference in the infection rates between people who drank alcohol versus those who did not drink alcohol as a risk factor for postoperative infections was not statistically significant, but it did approach significance (*P* = .05).

## Discussion

The results of this study show that the addition of vancomycin powder reduces postoperative SSIs. The addition of vancomycin powder to the wound prior to closure decreased postoperative infections from an incidence of 5.7% down to a rate of 2.0%. This difference was statistically significant (*P* = .018). The addition of tobramycin powder to the wound in addition to vancomycin further decreased the infection rate down to 1.8%. The postoperative infection rate was statistically significant (*P* = .041) when compared with the no-powder group. However, the difference was not statistically significant (*P* = 1.00) when compared with the vancomycin group.

There was a trend toward gram-negative organisms with the addition of more antibiotic powder. In the group with no powder, the organisms cultured were 66% MSSA and 33% gram-negative organisms. In the vancomycin group, 30% of the organisms cultured were *Staphylococcus aureus* and 60% gram-negative organisms. In the vancomycin and tobramycin powder group, 100% of the organisms cultured were gram-negative organisms. All these patients were successfully treated with irrigation and debridement and antibiotic treatment. The instrumentation did not need to be removed in any of the patients to successfully treat the infections.

Chi-square analysis was performed to see if there was a difference among the groups with regard to age, medical comorbidities, smoking, and alcohol use. There was no statistically significant difference between the groups with regard to these factors. A logistic regression analysis was also performed to see if these factors were independent risk factors for developing postoperative infections. In this study, smoking was found to be a risk factor for developing postoperative wound infection while age, alcohol use, and patients with medical comorbidities approached statistical significance as independent risk factors for developing postoperative wound infections.

SSIs pose significant problems to patients and surgeons. Patients must undergo additional procedures to eradicate the infection and this often results in additional pain, a longer recover time, and more days missed from work. These problems are even greater in patients who have posterior instrumentation placed. In patients who develop SSIs that have posterior instrumentation, there is the added risk of loss of correction if the instrumentation must be removed, decreased rate of fusion, and even the risk of osteomyelitis.^1–3^ Hence, it is to the patients’ and surgeons’ best interest to do everything that is reasonable to decrease the incidence of SSIs.

One of the strategies to reduce SSIs in patients that have posterior instrumented lumbar fusions is to add vancomycin powder to the wound prior to closure. There are many studies that show vancomycin powder reduces the incidence of SSIs.^10–13^ However, subsequent studies have further shown that SSIs still occur even with the addition of vancomycin powder. In a study by Hey et al,^14^ the authors performed a retrospective review on patients undergoing spine surgery. In the study, there were 117 patients who received vancomycin powder and 272 patients where no powder was added. The authors noted that there was a statistically significant reduction in the incidence of SSIs with the addition of vancomycin powder. But they also noted that the most common causative organism changed to *Pseudomonas aeruginosa* (35.2%). In another study by Ghobrial et al,^15^ the authors performed a retrospective review on 981 patients where vancomycin powder was placed in the wound intraoperatively. They noted an increased incidence of gram-negative or polymicrobial spinal infections with the addition of vancomycin powder. Hence, there are concerns that vancomycin powder may not be effective enough to counteract gram-negative organisms. So, this has led some surgeons to increase antibiotic use especially when spinal instrumentation is used. In a study by Salsgiver et al,^16^ the authors explored the use of cefazolin and tobramycin pre- and postoperatively to reduce infections in patients who had instrumented fusions for adolescent idiopathic scoliosis. The authors concluded that underdosing of either cefazolin or tobramycin resulted in an increased infection rate. In another study by Boorkhuu et al,^17^ the authors reported on using gentamicin-loaded allograft to decrease deep wound infection rates after spinal fusion in cerebral palsy patients. The authors found that the incidence of deep wound infection after spinal fusion in 220 children with cerebral palsy scoliosis decreased from 15% to 4% with the use of gentamycin-impregnated allograft bone. Hence, there has been growing interest in using more antibiotics in spine surgery to decrease infection rates.

This is the first study evaluating the use of intraoperative vancomycin and tobramycin powder together in the wound. The results of this study suggest that the addition of tobramycin does not reduce the infection rate any more than the addition of vancomycin alone. Although Watanakunakorn et al^18^ demonstrated vancomycin-tobramycin synergism in vitro, one reason why the clinical addition of tobramycin did not reduce infections may be dosing. We used a 1:1 ratio of vancomycin powder to tobramycin powder in pediatric patients and a 2:1 ratio in adult patients. Perhaps, the ratio needs to be tilted to favor tobramycin further in order to produce a meaningful effect. However, increasing the amount of tobramycin powder carriers its own risks, such as greater nephrotoxicity.

Also, there was a change in organisms cultured from the wound with the addition of more antibiotic powder. There was a change from primarily MSSA (66%) aureus in the no powder group to 60% gram-negative organisms when vancomycin is added. In the vancomycin and tobramycin powder group, 100% of the organisms cultured were gram-negative organisms. So, the use of antibiotic powder must be carefully considered in each case because there is the risk that we will ultimately select for more virulent organisms with the addition of more antibiotic powder.

There are many limitations to this study. First, this is only a 3-center study including only 5 surgeons. With a larger number of hospitals and more surgeons, the results may be more variable. Second, this is a retrospective review. With a prospective, randomized study it may be possible to stratify patients better. However, we did a chi-square analysis and there was not a statistically significant difference between the groups with regard to infection risk factors such as age, smoking, medical comorbidities including diabetes, and alcohol use. A larger sample size would also improve the power of this study; however, the number of subjects in this study was sufficient for statistical analysis.
